# Trust me; I know what I am doing investigating the effect of choice list elicitation and domain-relevant training on preference reversals in decision making for others

**DOI:** 10.1007/s10198-021-01283-3

**Published:** 2021-03-20

**Authors:** Sebastian Neumann-Böhme, Stefan A. Lipman, Werner B. F. Brouwer, Arthur E. Attema

**Affiliations:** Erasmus School of Health Policy and Management, Erasmus University Rotterdam, P.O. Box 1738, 3000 DR Rotterdam, The Netherlands

**Keywords:** Choice, Decision making for others, Preference elicitation, Preference imprecision, Preference reversals

## Abstract

One core assumption of standard economic theory is that an individual’s preferences are stable, irrespective of the method used to elicit them. This assumption may be violated if preference reversals are observed when comparing different methods to elicit people’s preferences. People may then prefer A over B using one method while preferring B over A using another. Such preference reversals pose a significant problem for theoretical and applied research. We used a sample of medical and economics students to investigate preference reversals in the health and financial domain when choosing patients/clients. We explored whether preference reversals are associated with domain-relevant training and tested whether using guided ‘choice list’ elicitation reduces reversals. Our findings suggest that preference reversals were more likely to occur for medical students, within the health domain, and for open-ended valuation questions. Familiarity with a domain reduced the likelihood of preference reversals in that domain. Although preference reversals occur less frequently within specialist domains, they remain a significant theoretical and practical problem. The use of clearer valuation procedures offers a promising approach to reduce preference reversals.

## Introduction

The elicitation of preferences, i.e. finding out if one prefers A over B or vice versa, is central in economics and, therefore, relevant to many topics studied in health economics, such as health state valuations, multi-criterion decision analysis [8], patient preferences [55], and studies on physician behaviour [38]. Many different methods are used to elicit preferences in the relevant target group, including well-known methods like willingness to pay [40], time trade-off (e.g. [24], and discrete choice experiments (e.g. [33]).

A disturbing finding is that different preference orderings may be obtained, especially when using different methods. This phenomenon is typically referred to as *preference reversal*. For example, people may prefer option A over B when directly asked to choose between them but have a higher willingness to pay for B than for A [34, 60]. To illustrate, imagine a person who, when given a direct choice, indicates that she prefers surgery over physiotherapy for a given condition. Given this observation, we would, *ceteris paribus*, expect her to also be willing to pay more (or at least not less) for surgery than for physiotherapy. If this is the case, her preferences could be classified as consistent. In practice, however, her willingness to pay for physiotherapy may turn out to be higher than that for surgery. This may be classified as inconsistent and constitutes a preference reversal. If such preference reversals occur, preferences may not be stable, but depend on and can reverse between different elicitation methods and procedures. As a result, it is no longer possible to determine which (if any) method yields ‘true’ preferences [17]. Hence, preference reversals offer substantial methodological challenges, but also form a general and fundamental problem to applied work and decision-making in health and other settings.

Unfortunately, preference reversals appear to be a robust phenomenon, which typically occurs when comparing preferences for risky outcomes elicited using different methods [56] or different operationalisations of the same method [6]. In a classic example, Slovic and Lichtenstein [47] offered subjects two risky lotteries, referred to as the P-bet and the $-bet. The former included a high chance of a moderate reward (e.g. 95% chance of winning 40$, or lose 10$ otherwise), while the latter involved a lower chance of a high reward (e.g. 15% chance of winning 160$ or lose 15$ otherwise). Preferences were first elicited using *direct choice*, i.e. subjects were asked to indicate which lottery they would choose. Next, subjects were asked to indicate the monetary values they would assign to both lotteries, i.e. their *valuation*. Slovic and Lichtenstein [47] found that for lotteries with similar expected values, subjects chose the P-bet over the $-bet, but assigned a higher monetary value to the $-bet compared to the P-bet. This finding has been replicated frequently (e.g. [36, 53, 56]) and constitutes a preference reversal, as economic theory predicts that the preferred lottery should also have been assigned a higher valuation.

By now, preference reversals have been studied extensively for monetary outcomes, using many different settings and methods (for a review, see: [56]). Preference reversals in decisions related to health outcomes have been documented in several studies as well [14, 49, 50, 52, 57]. To our knowledge, the only study directly comparing preference reversals in choices regarding health and money is that of Oliver and Sunstein [51], who found a higher rate of preference reversals for health. Given that preference reversals pose a significant methodological and practical problem, improving our understanding of causes and potential ways to reduce preference reversals in different contexts remains crucial. Hence, we report the findings of an experiment in which preferences were elicited in a sample consisting of both medical and economics students for both health and monetary outcomes. This experiment expands earlier work in two directions.

First, in the seminal work by Lichtenstein and Slovic [47], preference reversals were demonstrated by comparing direct choice and valuation, where the latter was obtained with open-ended questions. Subsequent work, instead, obtained valuations through choice-based procedures and has shown this reduces preference reversal [7, 10, 16, 42, 48]. Furthermore, Oliver [50] argued that people are unlikely to have fixed preferences for unfamiliar goods and may use unstable heuristics when asked to value them using open valuation. As a result, there have been attempts to simplify open-ended valuation elicitation for respondents. For example, Oliver [50] tried an assisted valuation procedure by presenting respondents a selection of amounts to pay for a risky operation but found no notable differences with open valuation. In this study, we continue this line of research by using *choice list elicitation* (as popularised by [41] for valuation. This choice-based method for preference elicitation is often applied in behavioral and experimental economics as it is easy to explain and implement [2].

Second, while some authors have explored preference reversals from the perspective of a social planer [9, 60], preference reversals in decisions on behalf of others have received little attention (see [50], for an exception). Investigating preference reversal in this area may be an important avenue for health economics research, as for many real-life decisions about health, one often has to rely on the advice and actions of others, e.g. physicians proposing preferred treatment options. Indeed, Arrow [4] identified the reliance on physicians’ expertise as one of the main reasons for a separate study of the economics of health. Similarly, one may also rely on experts in decisions about money, e.g. financial experts selecting investment portfolios. In both the health and monetary domain, the *outcomes* of decisions made by those with different or more expertise in a particular field have been extensively studied (e.g. [1, 15, 22, 27, 39, 46]). In this paper, instead, we extend this research by studying the *consistency* of decision-making, and by extension focus on an entirely new aspect of the preference reversal phenomenon: the consistency of those advising others inside (and outside) their field of expertise. In our experiment, consistency is tested with students from different disciplines, and throughout this paper, we will refer to any effects related to deciding in a domain relevant to their respective field of study as *domain-relevant training.*

Note that although some evidence exists suggesting that students and physiciancs have similar preferences [18], students are obviously still training to become experts. Besides their field of study, the two groups of students in our study may also differ in terms of skills and traits. For instance, those that precede and affect self-selection into different educational tracks, like the wish to help others in medical students (e.g. [29, 32]). Furthermore, earlier studies have aimed to implement a real patient benefit into the decision-making process to create real incentives, for example by transforming the patient health benefits into a monetary amount that is then donated to a charity [3, 18, 19, 39, 44, 45]. Our work instead uses hypothetical scenarios for *both* health and monetary decisions. This lack of incentive-compatibility may be seen as a limitation [30], but it enabled us to study preference reversals for decisions involving realistic stakes of moderate size in both domains (as in [51]). In particular, we aimed to describe a scenario that reflected the medicial decision context as realistic as possible. Converting the benefits in the scenarios to real health gains through donations to some health-related charity would likely result in very small and uncertain health gains, of a different nature than the ones studied here. This may also negatively affect the comparability between the two domains. Hence, also in order to prevent apparent procedural differences between health and money, preferences were elicited with hypothetical and relatively large and realistic stakes throughout the entire experiment.

The remainder of the paper is structured as follows; firstly, we will form hypotheses for our study. We then continue to explain our experimental procedure in the methods section and finish with presenting our results and discussing them in the context of the literature.

## Hypotheses for effects of choice list elicitation and domain-relevant training

Preference reversals are often explained by overpricing of the $-Bet (i.e. low chance to gain a high outcome) as a result of scale compatibility [59]. This hypothesis suggests that people focus on different aspects of lotteries depending on the elicitation method. In direct choice, they give more attention to probabilities, which benefits the P-Bet (i.e. the high chance of winning a moderate amount), as this bet has a higher chance of yielding a positive result. In valuation, operationalised by using open-ended questions (e.g. “For what price would you sell this lottery?”), subjects focus on the unit in which they should express their valuation. In the study by Tversky et al. [59], this focus on monetary amounts favours the $-Bet and therefore could explain the relatively high rates of preference reversals. If rather than open-ended questions, choice list elicitation is applied, both direct choice and valuation would involve choice. Seeing as earlier work has consistently shown that preference reversals are lower when valuation is choice based [7, 10, 16, 42, 48], we formed our first hypothesis (H1):

H1: The use of choice list elicitation will lead to fewer preference reversals.

Furthermore, it is well-known that preference elicitation (for risk) may contain noise or imprecision [12], which may be more likely if preferences are elicited for outcomes that one has no decision experience with or interest in. According to Butler and Loomes [20, 21], indicating the value of a risky gamble, such as a P-bet or $-bet (i.e. by providing a certainty equivalent) is a difficult task which leads to imprecision, and this imprecision may explain part of the systematicity of preference reversals. Hence, the relatively high rates of preference reversal observed in earlier studies on health outcomes [14, 49–51, 57], may partly be explained by the fact that most samples in these studies are generally unfamiliar with decisions about health. Indeed, Beshears et al. [11] indicate that a lack of experience and choice complexity increase the occurrence of decision-making errors in preference studies (such as preference reversals). Pinto‐Prades et al. [52] provided more support for the role of imprecision in producing preference reversals by showing how preference reversals for health outcomes can be reduced by repeating preference elicitations. Hence, domain-relevant training may reduce preference reversal by reducing such imprecision, as students through their (selection into) domain-relevant training may be more familiar with considering outcomes in one domain rather than another. Thus, our second hypothesis (H2) is:

H2: Participants with domain-relevant training will show fewer preference reversals in their area of expertise.

## Methods

### Sample and experimental design

**Fig. 1:**
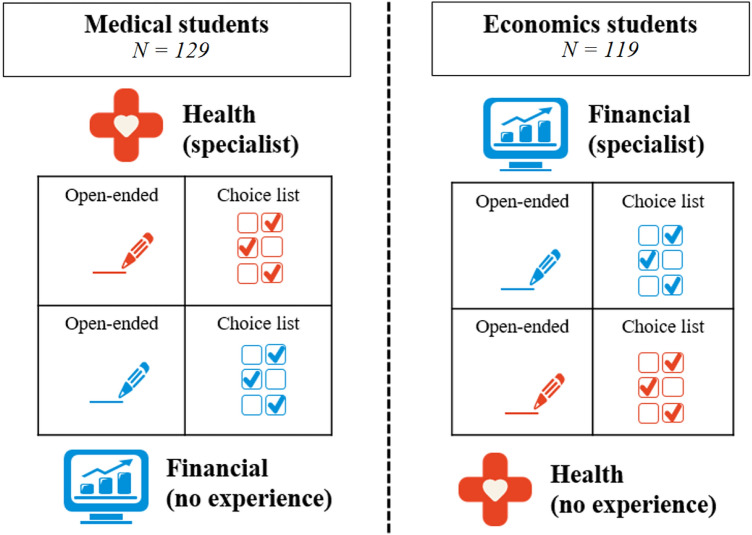
Survey design of the two domains and valuation procedures

To ensure that every participant had at least some prior experience with choices in one of the domains, we aimed to only recruit economics, business and medical students beyond their first year of studies. Several screening questions were in place, to avoid recruiting students that did not meet these conditions. Our full sample of 252 students was comprised of 129 medical students, 121 business and economics students (henceforth: economics students) and two other students (removed from the sample). Additionaly, two students were excluded who reported being in their first year of studies, yielding a final sample of 248 students. Recruitment of these students differed depending on their discipline. Economics and business studentswere recruited from the subject pool of the experimental laboratory at Erasmus School of Economics, while medical students were recruited through messages in the virtual learning environment of two University Medical Centres (in Rotterdam and Leiden). Subjects were paid a flat fee of 10 Euros (paid out as a gift voucher) for participating in the experiment. Both groups of students completed an online experiment, which was operationalised in Qualtrics Survey Software, with a two by two within-subjects factorial design applied in two samples, using the following two factors: i) outcome domain (health vs financial), and ii) valuation procedure (open-ended vs choice list).^1^ This design allows us to study preference reversals within-subjects in four blocks and allows between-subjects comparisons based on discipline (i.e. economics or medicine). An overview of our experimental design is provided in Fig. 1. To avoid ordering and learning effects the order of outcome domains and valuation procedures was randomised.

### Experimental procedure

The online experiment started with general instructions and a practice block (see Appendix A). Afterwards, participants completed a total of 12 questions eliciting their preferences for health and investment decisions (on behalf of others) with one choice and two valuation questions for each condition. Both scenarios began with an introduction page informing participants which role they would have in the experiment that followed. Graphical elements were added to inform respondents which type of question they were answering and to reduce the repetitiveness of the questions. After completing the 12 questions, demographics were collected. More specifically, we collected information on age, gender, statistical competency, and year of study (see Appendix B for an overview of questions used).

#### Eliciting preference reversals

The questions per condition all followed a similar structure, following the classic study by Slovic and Lichtenstein [47] : i) a strict choice between two risky lotteries with similar expected values (henceforth P-bet and $-bet), ii) valuation of P-bet, iii) valuation of $-bet (for an overview of P-bets and $-bets used, see Table 1). The order of these three questions was randomised within each condition. We recorded a preference reversal if a respondent chose the P-bet over the $-bet in the direct choice, but at the same time valued the $-bet strictly higher in the valuation question. This commonly observed reversal pattern is usually referred to as a ‘predicted preference reversal’, as it is predicted by scale compatibility [59]. Preferring the $-bet while assigning a strictly larger value to the P-bet is defined as an ‘unpredicted preference reversal’. We will interpret subjects indicating to prefer one bet in direct choice while assigning it a higher or equal value in valuation as having consistent preferences.

**Table 1:**
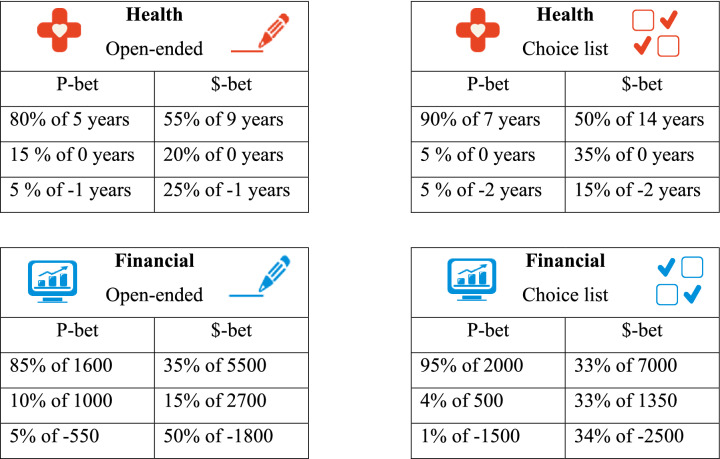
P-bets and $-bets used for health and financial outcomes in all four conditions

#### Operationalisation of outcome domains (health vs financial)

In both domains, respondents hypothetically advised a person on a decision between two risky prospects. In the financialscenario, respondents advised clients on how to invest their money in different portfolios. The health scenario involved recommending treatment options for a terminally ill patient, where the patient health status was described by using the dimensions of the EQ-5D instrument (see Appendix A for exact instructions). Whereas in the original set-up by Slovic and Lichtenstein [47] , which was extended to health outcomes by [49, 50], risky prospects were two-outcome mixed gambles (consisting of a gain and a loss), Table 1 shows that the P-bets and $-bets in this study used three outcomes. The third outcome was included to increase realism,^2^ as both investment and medical decisions typically have at least three outcomes: a gain (high return on investment or medical treatment is successful), ‘the status quo’ (moderate return on investment or medical treatment is unsuccessful), and a loss (portfolio value decreases or side-effects of medical treatments). In each question, graphical elements like those in Fig. 1 were used to emphasise (changes to) the outcome domain and valuation procedure being used.

#### Operationalisation of valuation procedure (open-ended vs choice list)

**Fig. 2:**
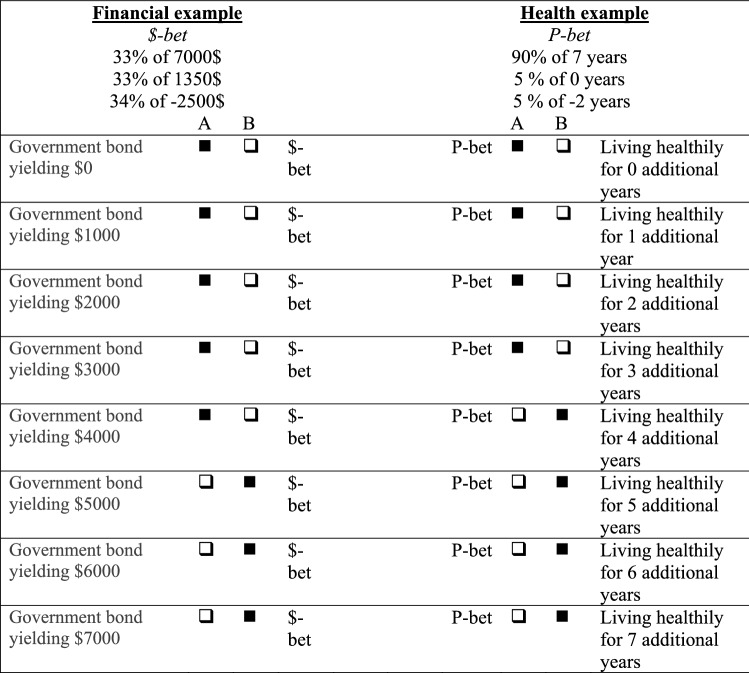
Hypothetical response to choice list valuation of a $-bet (financial) and P-bet (health), yielding certainty equivalents of 4500$ and 3.5 years, respectively

For health outcomes, open-ended valuation was operationalised as follows: students were instructed to compare the P-bet ($-bet) to a treatment yielding some amount of life years in perfect health for certain, where students were asked to provide the minimum amount of life years that would lead them to recommend patients to take this certain treatment over the P-bet ($-bet). For financial outcomes, the open-ended valuation was operationalised as follows: students were asked to compare the P-bet ($-bet) to a government bond yielding a sure gain and asked to indicate how large this gain should be for the bond to be equally good to the P-bet ($-bet). In both outcome domains, students were required to provide this certain amount of life years or money in an open answer field, i.e., students reported a certainty equivalent. Choice list valuation was operationalised by offering respondents a list of increasing amounts of money (in increments of 1000$, followed by a choice list in 100$ increments) or life years (in yearly increments) to choose from. Figure 2 shows an example of such a choice list valuation procedure for valuation of a P-bet, where at some point students switch from preferring the P-bet to a certain outcome. As is usual in choice list methodology [41], the certainty equivalent is obtained by taking the average of the certain outcome above and below the switching point (see Fig. 2 for examples). This procedure was guided as the choice lists were programmed to prohibit multiple switching points and choices that violated dominance.

## Results

### Descriptive statistics

Sample characteristics for these two groups of students can be found in Table 2. Comparisons between the two groups yielded some significant differences, showing that economics students (relative to medical students) were more likely to be male, and reported being in a higher study year and more competent in statistics.

**Table 2:** Sample characteristics by study discipline

	Economics (*n* = 119)		Medicine (*n* = 129)		Total (*n* = 248)		Econ vs. Medical
	Mean	Std. dev.	Mean	Std. dev.	Mean	Std. dev.	
Age	21.60	1.94	21.43	2.24	21.51	2.10	
Stat. comp.	2.94	1.02	2.51	0.82	2.72	0.94	*p* < 0.02
Study year	3.81	1.32	3.58	1.69	3.69	1.53	*p* < 0.02
Gender	Female 58	Male 61	Female 104	Male 25	Female 162	Male 86	*p* < 0.002

1 indicating “I had no statistical training”, 2 “I feel somewhat competent with statistics”, 3 “I know my way around statistics, but I'm not an expert”, 4 “I feel competent in statistics”, 5 “My specialization is statistics”.

Preference reversals for each scenario were first analysed descriptively by creating a dummy variable, which indicated if a preference reversal occurred or not. Table 3 shows the overall results of this online experiment, which indicate that predicted preference reversals were the most occurring combination of preferences in all conditions. Furthermore, only very few unpredicted preference reversals occurred, representing just over 1% of all combinations of preferences. Hence, we will study both reversals combined, and for brevity refer to these as ‘*the rate of preference reversal’*.

**Table 3:** Overall frequency distribution for combinations of preferences per condition, observations and (%)

Pattern	Health		Financial		Inter-pretation
	Open-ended	Choice list	Open-ended	Choice list	
P$	147 (59.3%)	120 (48.4%)	137 (55.2%)	94 (37.9%)	Predicted reversal
$P	0 (0.0%)	3 (1.2%)	1 (0.4%)	3 (1.2%)	Unpredicted reversal
PP	77 (31.0%)	89 (35.9%)	82 (33.1%)	85 (34.3%)	Consistent
$$	24 (9.7%)	36 (14.5%)	28 (11.3%)	66 (26.6%)	Consistent

The pattern P$ indicates that the P-bet was chosen in the choice task, but that the $-bet was valued strictly higher in the valuation task, while $P indicates the reverse pattern. PP and $$ indicate a choice for a bet that was valued at least as good or higher (i.e. no inconsistency)

### Comparisons by students’ discipline, outcome domain, and valuation procedure

We compared preference reversals by study discipline, outcome domain and valuation procedure using chi-squared tests. When we sum preference reversals (i.e. predicted and unpredicted), we find that combined for all conditions, fewer reversals occurred in the financial domain than in health, economics students show fewer reversals than medical students and fewer reversals occur when choice lists are used compared to open valuation (see Table 4).

**Table 4:** Reversals rates by domain, training and procedure

	Domain	Health	Financial	$\chi^{2}$
	Rate of reversal	54.4%	47.6%	*p* < 0.05

	Training	Medicine	Economics	$\chi^{2}$
	Rate of reversal	56.6%	45.1%	*p* < 0.001

	Procedure	Open Val	Choice list	$\chi^{2}$
	Rate of reversal	57.5%	44.4%	*p* < 0.001

When comparing rates of preference reversals between-subjects (see Table 5), we note that for open valuation, an effect of domain-relevant training appeared to occur. Economics students had a significant 14.6 pp difference between financial and health outcomes using open valuation (9.8 pp using choice lists) and were, as expected, more consistent in the financial domain (their area of expertise).

**Table 5:** Reversal rates between subjects

Economics students				Medical students			
Rate of reversal	Open Valuation	Choice List	$\chi^{2}$(method)	Rate of reversal	Open Valuation	Choice List	$\chi^{2}$ (method)
Health domain	59.3%	43.1%	*p* < 0.05	Health domain	59.7%	55.0%	*p* = 0.450
Financial domain	44.7%	33.3%	*p* < 0.10	Financial domain	65.9%	45.7%	*p* < 0.05
$\chi^{2}$ (domain)	*p* < 0.05	*p* < 0.05		$\chi^{2}$ (domain)	*p* = 0.303	*p* = 0.135	

Using choice list valuation, both economics and medicine students were more consistent compared to open valuation (i.e., showing lower rates of preference reversal). The most substantial reductions in the rate of preference reversals through choice lists could be observed outside of the respondent’s area of expertise. The rate of preference reversals of economics students using choice lists was 16.2 pp lower in the medical domain as opposed to an 11.4 pp reduction in the financial domain. Medical students showed a non-significant 4.7 pp reduction in the rate of preference reversals in the health domain and a significant 20.2 pp reduction in the financial domain when preferences were elicited with choice lists.

To substantiate our descriptive findings further, we ran a logistic mixed-effects regression, which allowed us to determine to what extent the chance of observing a preference reversal was affected by our experimental conditions. Table 6 shows the results for a logistic regression model with random subject effects and fixed effects for a) domain (financial vs health), b) discipline (economics vs medical students), c) procedure (choice list vs open-ended valuation, d) domain-relevant training (domain × discipline interaction) and e) interaction term for procedure and discipline. These analyses showed that preference reversals are more likely to occur a) in the health domain, b) for decisions by medicine students, and c) for open valuations (as opposed to choice list elicitation). Furthermore, we observed a marginally significant interaction between discipline and domain (i.e., the effect of domain-relevant training): medical students were less likely to show preference reversals in their ‘own domain’. Importantly, when exploring the robustness of our findings, we found that our main findings were mostly unaffected by controlling for demographics and order effects. The results of these analyses can be found in Appendix C.

**Table 6:** Results of logistic mixed-effects regression predicting the preference reversal by our experimental conditions

	Estimate	*SE*	*Z*	*p*
Constant	− 0.84	0.19	− 4.56	** < 0.001**
Main effects				
Discipline (medical)	0.79	0.25	3.36	**0.001**
Domain (health)	0.59	0.20	2.99	**0.003**
Procedure (open ended)	0.63	0.20	3.18	**0.001**
Interaction effects				
Domain-relevant training (medical × health)	− 0.52	0.27	− 1.91	*0.06*
Discipline (medical) × Procedure (open)	− 0.10	0.27	− 0.38	0.71

Bold-faced *p*-values are significant at *α* = 1%, italicized *p*-values are significant at *α* = 10%

## Discussion

This study investigated whether domain-relevant training, gathered through selecting into and exposure to education to become a physician or economist, and choice list elicitation procedures reduced the rate of preference reversal in decision making for others for both health and money. Given that we studied preference reversals for both health and financial outcomes, the results of this study can be compared to the extant literature in these two domains. Overall, we find preference reversals to occur frequently with strictly reversed preferences occurring in 32–66% of the sample, depending on the condition. These high rates of (predicted) preference reversals are in accordance with earlier studies for financial outcomes [34, 47] and health [49–51, 57]. Some studies, often with designs that deviate more from the original set-up used by Lichtenstein and Slovic [47], find somewhat lower rate rates of preference reversals – especially for health (e.g. [14]). Oliver and Sunstein [51] compared preference reversals for health and money (and other domains) using different samples for each domain and found higher overall rates of preference reversal for health, which we confirmed in our study with direct within-subjects comparisons. Furthermore, for three out of four between-subjects comparisons, preference reversals occurred more frequently for health.

In addition, our design allowed comparing open-ended valuations and computer-assisted choice lists. The latter has only recently been introduced in preference elicitation in health economics (e.g. [3, 5, 28, 43, 52]). In line with our first hypothesis, we found that choice-based valuations, using guided choice list elicitation, reduced the rate of preference reversals for both health and money. Hence, our findings confirm earlier work for health [7] and money [10, 16]. Moreover, it appears that choice lists yield a lower rate of preference reversals when they are used in a domain that is unfamiliar to the respondent. This would make choice lists elicitation especially attractive for preference elicitation in general population samples where no experience with the outcome domain can be expected.

Furthermore, we find a higher rate of preference reversal for medical students overall, and a trend suggesting that the increase in rates of preference reversals from money to health is smaller for medical students (as shown by the regression results in Table 6). For example, when medical students completed the open-ended valuation, we found fewer preference reversals for health than for financial outcomes, but not when using choice lists. This effect was stronger for economics students, who had a lower rate of preference reversal in the financial than in the health domain in both methods. Therefore, we find some support for our second hypothesis, that subjects with domain-relevant training show fewer preference reversals in their respective area of expertise.

Overall, we found a more substantial effect of valuation procedures as opposed to domain-relevant training. This may suggest that in our study scale compatibility [59] plays a larger role in generating preference reversals than imprecise preferences [21]. The fact that controlling for the years of education of respondents did not affect our findings is in line with this (see Appendix C). However, this experiment was unable to provide conclusive evidence regarding this issue, as we used a between-subjects design to test for domain-relevant training (as opposed to studying one individual accumulating experience). This distinction may be important, because even though economics and medicine students may differ in the content of their experience, they may also differ in terms of experience with participating in preference-based experiments. Hence, the higher overall rates of preference reversal we observed for medical students may also be a reflection of imprecise preferences due to the unfamiliarity or a lack of domain-relevant training in participating in experiments, providing support for the conjecture of Butler and Loomes [21]. Furthermore, while this study allowed us to test if the consistency in choices is affected by the elicitation procedure and the familiarity with the outcome domain, we have no way of determining what the ‘true preferences’ of participants would be. Moreover, we cannot assert that observing fewer preference reversals implies that elicited preferences are more aligned with such ‘true preferences’.

Regardless of our attempts to reduce them, preference reversals remained prevalent. Earlier work provides several explanations for these findings. First, as has been shown by Pinto‐Prades et al. [52], choice list elicitation is a transparent and straightforward way to elicit preferences. This explicit transparency may have allowed subjects to deduce that the goal of this task was to observe an indifference between two outcomes. If respondents are aware of the goal of the task, this could lead to strategic choices or influences from previous choices (a consistency that does not necessarily imply more precise estimates of preferences). Other methods, e.g. the hidden choice-based procedure developed by Fischer and colleagues [26], reduce these influences by spreading elicitations over multiple items that occur in random order, and they have been shown to reduce the rate of preference reversals [26, 52].

Second, we opted to study preference reversals in decisions for others, as this is relevant in real life and in the context of economics and medicine students’ training. Oliver [50] found that preference reversals occur more frequently in the context of social decision making. In our experiment respondents advise others on decisions and, hence, one might object to referring to these choices on behalf of others as ‘preferences’ (and inconsistencies as ‘preference reversals’). However, similar to Oliver [50], we decided to also use the established term ‘preference reversal’ in a context of decision making for others, since the phenomenon is well established under this term in the literature, although it needs noting that in doing so, we use the term preference in a broad sense.

Third, this experiment was completed using online survey software. Although several studies found little differences between lab and online studies [13, 23, 31, 54], other studies found that completing research in online environments may lead to higher variances or more noise (e.g. [61]. In our study, more noise would have been reflected in higher rates of preference reversals, both predicted and unpredicted. Given that the number of unpredicted preference reversals was negligible (less than 1.5%), our results give a little indication to expect a large effect of noise related to the online nature of the experiment.

Fourth, the recruitment procedures for the two groups of students differed between medical and economics students, but both groups were unaware of the nature of the experiment until they started it. Therefore, we expect the effect of this difference to be small. Self-selection into the experiment may hamper the generalizability of our findings, as this may involve a biased sample of students.

Finally, related to the issue of generalizability, our (relatively limited) sample comprised of 248 students of economics and medicine, which also raises the question whether our findings generalise to i) the general public, ii) other trained professionals and their respective domains, and iii) actual medical professionals or economists. Given the main dimensions on which our sample differed from the general public (e.g., age, education level and wealth), which are related to risk attitudes [35, 37], investigating the effects of choice-based elicitation in a general public sample would be an interesting venue for future research. Larger sample sizes would then also be more feasible to obtain. Furthermore, although recruitment may be time-consuming, to further study the effect of domain-relevant training on preference reversal, future work could recruit respondents working as trained experts in these fields, such as investment bankers (as in [1] or physicians (as in [18]). Although these studies give no indication to expect qualitatively different decision-making, such future work could explore if the positive trend related to domain-relevant training is amplified when more decision experience is accumulated.

## Conclusion

If observed preferences indeed depend on the way they are elicited, as we showed in this study, this is problematic. As long as revealed and stated preferences remain a cornerstone of research in health economics, such preference reversals offer a challenge to both empirical and theoretical work. Whereas preference reversals appear to be robust, occur frequently and are especially prevalent in unfamiliar domains, we believe this study may still offer some guidance for preference elicitation in research and practice in the future. First, guided choice-based valuation, such as choice list elicitation, may be a promising tool to obtain more consistent preferences. Whether this also implies a more accurate measurement of preferences remains to be seen. Second, although preference reversals were more common for decisions about health as opposed to money, we found that medicine students show fewer reversals in their own domain. This effect could have several explanations, but a positive interpretation would be that domain-relevant training improves consistency.

## Appendix A: Screenshots of the experiment (instructions and choice options)

### General instructions

Thank you for participating in this survey on decision-making about health and money. The goal of this study is to understand how people make choices for others for both financial decisions and when deciding for patients. Although the choices you will be making are hypothetical, please answer as if they were real. At the end of the experiment, you will receive a code, with which you can redeem your compensation for this study!

### Practice choice question

Please assume a patient has been diagnosed with a terminal condition with an expected survival of 1 year. There are two treatments that can extend the patient's life:

#### Treatment 1

85% chance of living healthy for 15 years.

15% chance of dying during treatment.

#### Treatment 2

60% chance of living healthy for 20 years.

40% chance of dying during treatment.

Here we would like you to select a treatment that you would recommend as the best option for the patient. There is no option of choosing neither treatment because this would result in the death of the patient due to the disease. Also, there is no right or wrong answer, we are just interested in your preferences between these treatments.

### Practice valuation question

A patient has been diagnosed with a terminal condition with an expected survival of 1 year. There are two treatment options.

#### Treatment 1

70% chance of living healthy for 8 years.

30% chance of dying during treatment.

#### Treatment 2

100% chance of living healthy for X Years.

What is the minimum amount of X (life years) you would require from treatment 2 to be willing to recommend it over treatment 1?

This is a hypothetical question because in health care any type of treatment involves risks. Here we would like to know at which point you are indifferent between the risky (treatment 1) and the certain treatment (treatment 2) so that you would regard them as equally good.

Some persons might consider a 70% chance to gain 8 life years (treatment 1) to be better than gaining 2 years without any risk (treatment 2), but they would consider both treatments equally good if the patient would gain 5 years for certain from treatment 2. In this case, the answer to the question would be 5.

I would recommend Treatment 2 for when the minimum amount of X life years is:

### Health domain—Direct choice

Health domain—Choice list valuation procedure

## Appendix B: Demographics questionnaire

The following questions were included to measure the demographics of our student sample.

1. What is your gender?
  - Female
  - Male
2. What is your highest educational degree?
  - No degree
  - Vocational training / apprenticeship
  - Secondary education diploma (HAVO/VWO)
  - Bachelor degree
  - Master degree
  - PhD
3. What is your field of study
  - Economics and related subjects (e.g. Econometrics, Health Economics, etc.)
  - Business Administration and related subjects
  - Medicine/Medical Science
  - Other
4. How would you rate your competence in statistics?
  - I had no statistical training
  - I feel somewhat competent with statistics
  - I know my way around statistics, but I’m no expert
  - I feel competent in statistics
  - My specialisation is statistics
5. In which year of your studies (starting from the Bachelor) are you?
6. In which country were you born?
7. How old are you?

## Appendix C: Robustness checks—logistic regression results

In this Appendix, we report additional regression results that illustrate that our main results are mostly unaffected by controlling for sample characteristics as well as order effects. We ran a series of mixed logistic regression models for which the results are reported in Table

**Table 7:** Fixed-effect estimates (with SE in brackets) for mixed-effects logistics regression analyses

Model	1	2	3	4	5
Main effects					
Discipline (medical)	**0.84 (0.25)**	**0.80 (0.25)**	*1.17 (0.62)*	**0.82 (0.25)**	**0.81 (0.25)**
Domain (health)	**0.59 (0.20)**	**0.59 (0.20)**	**0.59 (0.20)**	**0.59 (0.20)**	**0.59 (0.20)**
Procedure (open ended)	**0.63 (0.20)**	**0.62 (0.20)**	**0.62 (0.20)**	**0.63 (0.20)**	**0.64 (0.20)**
Interaction effects					
Domain-relevant training	− *0.52 (0.27)*	*− 0.52 (0.27)*	*− 0.52 (0.27)*	*− 0.52 (0.27)*	*− 0.52 (0.27)*
Discipline (medical) × Procedure (open)	− 0.10 (0.27)	− 0.10 (0.27)	− 0.11 (0.27)	− 0.10 (0.27)	− 0.10 (0.27)
Sample characteristics					
Age		0.05 (0.07)	0.06 (0.07)		
Statistical competency		0.07 (0.10)	0.14 (0.14)		
Study year		− 0.12 (0.09)	− 0.10 (0.10)		
Gender (male)		− 0.28 (0.18)	*− 0.48 (0.23)*		
Sample characteristics: interaction					
Statistical competency × Discipline (medical)			− 0.12 (0.19)		
Study year × Discipline (medical)			− 0.06 (0.11)		
Gender (male) × Discipline (medical)			0.47 (0.36)		
Order effects					
Domain order (health first)				− 0.17 (0.16)	− 0.08 (0.21)
Procedure order (valuation first)				0.13 (0.15)	0.23 (0.21)
Order effects: interaction					
Domain order (health first) × Procedure order (valuation first)					− 0.19 (0.30)

Bold-faced estimates are significant at *α* = 1%, italicized *p*-values are significant at *α* = 10%

7. Each model was similarly defined as the model reported in Table 4, which will be referred to as Model 1 in this Appendix, with additional fixed effects added as detailed below. We report the results for the following models:

- Model 2 (Sample characteristics): fixed effects for age, statistical competency, year of study and gender.
- Model 3 (Sample characteristics with discipline interaction): additional fixed effects for sample characteristics that differed significantly between study disciplines, i.e. statistical competency, year of study and gender.
- Model 4 (Order effects): fixed effects for domain order (health first vs. financial first) and procedure order (choice list first vs. open valuation first).
- Model 5 (Order effects with interactions): additional fixed effects for domain and procedure order interaction.

Note that because of the modest sample size of this experiment we ran models with main effects and interaction effects separately, as our study may not be powered to test for the latter.

Only the introduction of interaction terms with sample characteristics slightly affected our conclusions, as the effect of students’ discipline was now marginally significant (i.e. *p* < 0.10) rather than significant at *α* = 1%. It appears that part of this effect is driven by the difference in gender composition of our samples, as after controlling for this difference the effect of gender was marginally significant (*p* < 0.10). This suggests that (ceteris paribus) males were less likely to report a preference reversal.

## Footnotes

1 We also piloted a condition aimed at reducing preference reversals by using natural frequencies to communicate risks, but due to a programming error this data could not be included.

2 To check the realism of our P-bets ($-bets) and the instructions used for medical decision-making, we consulted a physician. Some minor changes were made to the framing (e.g. we increased the age of the patient whom students are to imagine they would be advising).
